# A novel methionine metabolism-related signature predicts prognosis and immunotherapy response in lung adenocarcinoma

**DOI:** 10.18632/aging.204687

**Published:** 2023-05-02

**Authors:** Qing-Hua Chang, Yuan-Cui Zhang, Dong-Ying Zhang, Ting Mao, Ran Chang, Nan Wang, Yun Ye, Zi-Jun Xu

**Affiliations:** 1 Department of Respiratory Medicine, The Affiliated Third Hospital of Jiangsu University, Zhenjiang, China; 2 Department of Radiology, The Affiliated Third Hospital of Jiangsu University, Zhenjiang, China; 3 School of Basic Medical Sciences, Fujian Medical University, Fuzhou, China; 4 Department of Nursing, The Affiliated Third Hospital of Jiangsu University, Zhenjiang, China; 5 Laboratory Center, Affiliated People’s Hospital of Jiangsu University, Zhenjiang, China

**Keywords:** methionine metabolism, lung adenocarcinoma, tumor microenvironment, molecular subtype, immunotherapy

## Abstract

Recent research revealed methionine metabolism as a key mediator of tumor initiation and immune evasion. However, the relationship between methionine metabolism and tumor microenvironment (TME) in lung adenocarcinoma (LUAD) remains unknown. Here, we comprehensively analyzed the genomic alterations, expression patterns, and prognostic values of 68 methionine-related regulators (MRGs) in LUAD. We found that most MRGs were highly prognostic based on 30 datasets including 5024 LUAD patients. Three distinct MRG modification patterns were identified, which showed significant differences in clinical outcomes and TME characteristics: The C2 subtype was characterized by higher immune score, while the C3 subtype had more malignant cells and worse survival. We developed a MethScore to measure the level of methionine metabolism in LUAD. MethScore was positively correlated with T-cell dysfunction and tumor-associated macrophages (TAMs), indicating a dysfunctional TME phenotype in the high MethScore group. In addition, two immunotherapy cohorts confirmed that patients with a lower MethScore exhibited significant clinical benefits. Our study highlights the important role of methionine metabolism in modeling the TME. Evaluating methionine modification patterns will enhance our understanding of TME characteristics and can guide more effective immunotherapy strategies.

## INTRODUCTION

In recent years, there has been a resurge of interest in cancer metabolism, which is now an established hallmark of cancer [1]. As first described by Otto Warburg, cancers preferentially use aerobic glycolysis for energy generation (now termed the “Warburg effect”) [2]. Besides aerobic glycolysis, multiple cancers have been shown to be highly dependent on methionine, an essential amino acid in one-carbon metabolism [3, 4]. Methionine is primarily metabolized to S-Adenosylmethionine (SAM), which donates the methyl group to DNA, RNA, and histones and is subsequently converted to S-adenosylhomocysteine (SAH) [5]. Although dietary methionine restriction has been shown to inhibit tumor growth profoundly and induce sensitivity to anti-cancer agents [6], the underlying mechanisms are poorly understood. It is possible that the altered methionine metabolic networks substantially influenced the epigenetic state of cancer cells, which ultimately led to transcriptional programming that favored tumor formation [6].

Recently, it was found that methionine-related metabolites were strongly enriched in patient-derived lung tumor-initiating cells (TICs) as compared with isogenic differentiated cells. Methionine restriction severely disrupted the tumor-forming ability of TICs, presumably by changing histone methylation levels. In addition, the study demonstrated the overexpression and tumor-promoting potential of two key methionine regulators (Methionine adenosyltransferase 2A [*MAT2A*] and Methylenetetrahydrofolate Reductase [*MTHFR*]) in lung adenocarcinoma [7]. Interestingly, two recent studies have also identified a causal link between tumor methionine metabolism and T cell immunity in the tumor microenvironment (TME) [8, 9], revealing cancer methionine signaling as a promising immunotherapeutic target.

It is worth noting that lung cancer remains as the leading cause of cancer death in 2022 [10], and the most common subtype is lung adenocarcinoma (LUAD) [11]. Emerging evidence showed that the application of immune-checkpoint blockade has offered a new therapeutic opportunity for advanced non-small-cell lung cancer (NSCLC) patients, for whom the treatment provides durable responses and improved long-term survival [12–18]. Therefore, it would be of great interest to decode the role of methionine-related molecules in LUAD and to understand the relationship between these genes and the TME. However, the aforementioned research involved only two methionine regulators, a comprehensive picture of methionine regulation in LUAD is urgently needed.

In this study, we comprehensively evaluated the expression profiles and genomic alterations of 68 methionine-related genes (MRGs) in LUAD. We also analyzed the prognostic value of MRGs in 30 datasets including 5024 LUAD patients. We identified three distinct MRG modification patterns with significant differences in clinical and immunological characteristics. In addition, we constructed a scoring system (MethScore), which was found to be related to the patient prognosis and immune responses of LUAD. We also found that this scoring system could predict the response to immunotherapy in lung cancer patients. Overall, our integrative analysis of MRGs in LUAD will provide new insights for tumor biological research.

## MATERIALS AND METHODS

### LUAD dataset source and preprocessing

**Figure 1:**
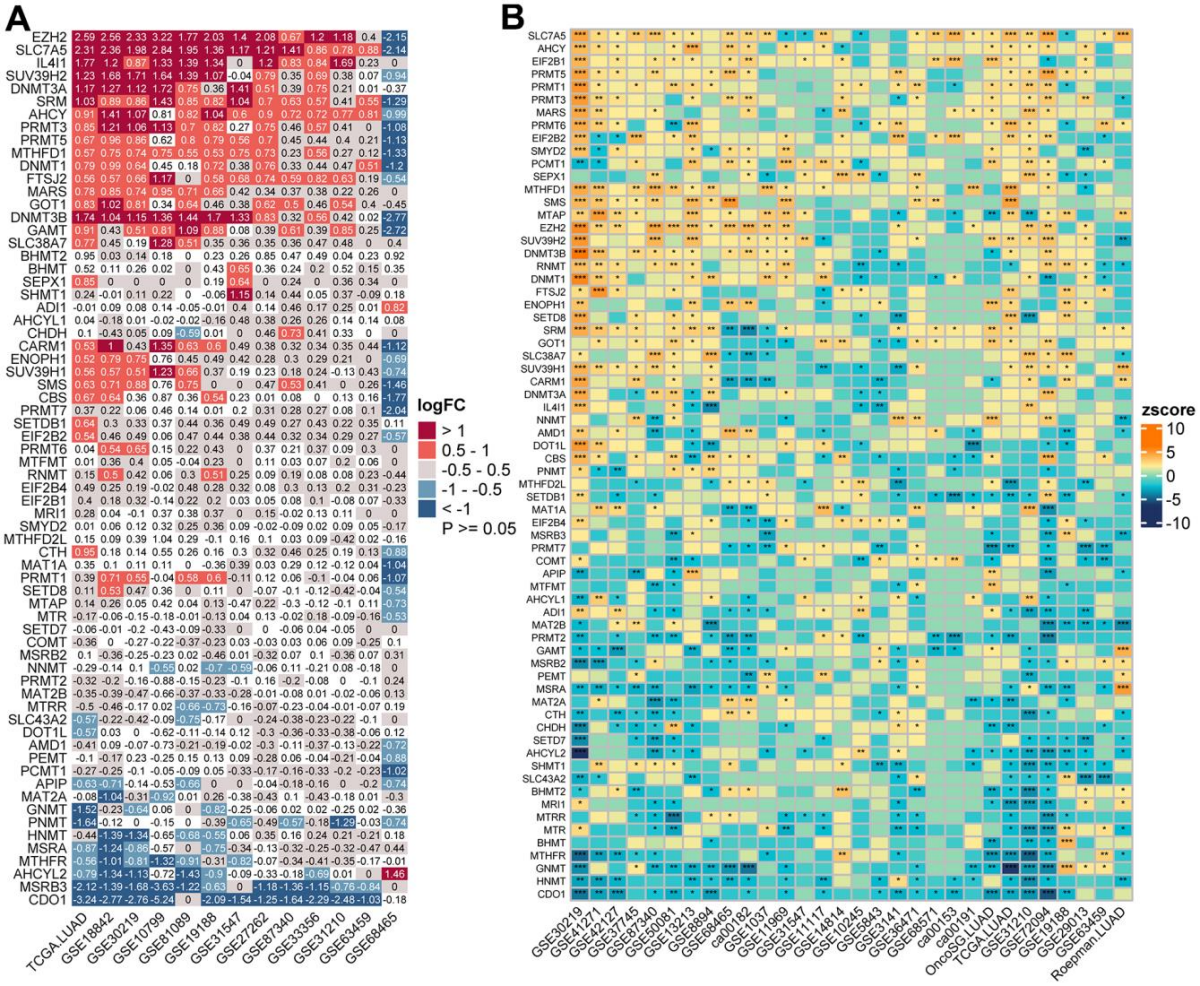
**Transcriptome-based expressional alterations and prognostic impacts of MRGs across multiple LUAD datasets.** (**A**) Heatmap showing the difference of mRNA expression levels of 68 MRGs between normal and LUAD samples in 13 datasets with matched controls. The color depicts the log2-transformed fold change (Log2FC) between tumor and normal tissues. Please also refer to Supplementary Table 2. (**B**) Association between *MRGs* expression and patient prognosis across 30 LUAD datasets with survival information as determined by the Log-rank test. The color represents Zscore-transformed hazard ratio (HR) and asterisks represent the statistical p value (*p < 0.05; **p < 0.01; ***p < 0.001). Please also refer to Supplementary Table 3.

Multi-omics data of LUAD, including mRNA (RNAseq), copy number variation (CNV), and single nucleotide variation (SNV) data, were downloaded from UCSC Xena Browser (https://xenabrowser.net). In addition, 38 LUAD gene-expression datasets and clinical annotation were retrieved from Gene-Expression Omnibus (GEO), cbioportal (https://www.cbioportal.org, OncoSG LUAD RNAseq data) and PRECOG (PREdiction of Clinical Outcomes from Genomic profiles; https://PRECOG.stanford.edu) database. The detailed clinical information of all eligible LUAD datasets was summarized in Supplementary Table 1. Thirteen datasets with matched controls were used to assess the differential expression patterns of MRGs (Supplementary Table 2 and Figure 1A). Thirty LUAD cohorts (including TCGA LUAD) with survival information were gathered to determine the prognostic value of MRGs (Supplementary Table 3 and Figure 1B). For microarray data that were not normalized, they were log2 transformed and then converted to a Z score. For microarrays that were already log2 transformed, the normalized matrix was Z-score transformed. For RNAseq data, the FPKM values were transformed into transcripts per kilobase million (TPM) values. Batch effects between arrays were corrected using the “ComBat” method from the sva package.

### Consensus clustering analysis of MRGs

Sixty-eight MRGs were retrieved from the Molecular Signatures Database (MSigDB, http://www.broad.mit.edu/gsea/msigdb/), Gene Ontology (GO) database, and previous articles and reviews [6, 9, 19–21]. The full list of these genes was shown in Supplementary Table 4. After median centering the expression values of MRGs, consensus clustering was applied using the ConsensusClusterPlus R package with partitioning around medoids (PAM) algorithm, euclidean distance, and 100 subsamples with a 0.8 random gene fraction. To investigate the differences in biological processes between different MRG subtypes, gene set variation analysis (GSVA) was performed with the Kyoto Encyclopedia of Genes and Genomes (KEGG) gene set (c2.cp.kegg.v7.4) retrieved from the MSigDB database.

### Differential gene expression analysis and functional enrichment analysis

We used the limma package to find differentially expressed genes (DEGs) among three MRG clusters. DEGs for each comparison were intersected to determine the final core gene set, which was visualized as Venn diagram using the R VennDiagram package. GO analysis and KEGG pathway analysis of the core gene set were performed using the clusterProfiler R package.

### Immune response analysis

TME characteristics including immune score, stromal score, and tumor purity were evaluated using the xCELL and ESTIMATE algorithm. In addition, the activities of a list of TME-related signatures were calculated using the single-sample gene set enrichment analysis (ssGSEA) algorithm, as implemented in the IOBR package [22]. The relative abundances of immune cells in LUAD patients were estimated using six deconvolution methods (CIBERSORT, EPIC, MCP-counter, quanTIseq, TIMER, and xCell), which have been integrated as a unified function “deconvo_tme” in the IOBR package. We compared the activity of TME-related signatures between each molecular subtype and the remaining samples. Then, the average fold changes (FCs) and Bonferroni-adjusted p-values (false discovery rate [FDR]) were computed using Wilcoxon rank sum test. The FDR values were categorized into six groups based on significance cutoffs for visualization (0.05, 0.01, 0.001, 1e-5, 1e-16). The pheatmap package was used to visualize the relative abundance of immune cells among groups. The TIDE (http://tide.dfci.harvard.edu/) was used to calculate T cell dysfunction, T cell exclusion, and other immune signature scores.

### Analysis of single-cell RNA-sequencing (scRNA-seq) data of LUAD

For single cell RNA-seq (scRNA) data analysis, three published scRNA-seq data of LUAD (EMTAB6149, GSE117570, and GSE127465) were downloaded from Tumor Immune Single-cell Hub (TISCH, http://tisch.comp-genomics.org/), a scRNA-seq database facilitating the exploration of TME across multiple cancer types [22]. Data were processed and visualized following the standard workflow of the single-cell data processing R package, Seurat [23]. Briefly, the downloaded h5 format was converted to a Seurat object using the “Read10X_h5” function. Low-quality cells were identified and filtered with a mitochondrial gene ratio >15% and with <500 or >4000 genes detected. The count matrix of high-quality cells was then subjected to normalization, scaling, principal component analysis (PCA), and clustering according to the Seurat tutorial. Cell clusters were annotated based on the downloaded cell metainformation table. The methionine metabolism activities for each single cell were computed using the scMetabolism package [24], and cell samples were categorized into high and low activity groups based on median activity levels.

### Collection and analyses of immunotherapy datasets

We collected two independent immunotherapy cohorts of lung cancer, including one cohort treated with anti-PD1 (Prat_CancerRes_2017, GSE93157) [25] and another cohort treated with anti-PD-1 (Cho_ExpMolMed, GSE126045) [26], to determine difference in immune checkpoint blockade (ICB) responsiveness between patients with high and low meth score. The response information was downloaded from the Supplementary Data of the respective papers.

### Construction of the methionine-related methscore

The methscore was calculated to quantify methionine metabolism of individual LUAD samples. First, the DEGs among three MethClusters were subjected to univariate Cox regression analysis to identify those significantly associated with OS. Then, the MethScore was calculated as the sum of PC1 and PC2 of the five core MRGs. Based on the median score, patients were divided into high and low MethScore groups. In addition, we also used the ssGSEA method to calculate and validate the value of MethScore.

### Statistical analysis and bioinformatics

Survival analysis was conducted using the “survival” and “survminer” packages. The optimal cutoff point of MethScore was determined by maximally selected rank statistics implemented in the “survminer” package. Survival probabilities of patient groups were estimated by the Kaplan-Meier method and compared with log-rank test. We used Wilcoxon rank-sum test to compare differences between two groups. Correlation between two continuous variables was assessed with Spearman’s rank correlation test. The R/Bioconductor package Maftools was used to compute the tumor mutation burden (TMB), generate oncoprint plots, and evaluate differentially mutated genes between two cohorts. Visualization was performed using the following R packages: “ggplot2”, “ggsci”, “ggpubr”, “RCircos” (for circos plots), “igraph” (for network plots), and “factoextra” (to visualize principal component analysis [PCA]). All statistical tests were two-sided, and a p-value less than 0.5 was considered to indicate statistical significance.

### Data availability

The datasets supporting this study are from previously reported studies and datasets, which have been cited. The data are available in the following open access repositories: TCGA, https://portal.gdc.cancer.gov/; UCSC Xena, https://xena.ucsc.edu; cBioPortal, https://www.cbioportal.org; GEO, https://www.ncbi.nlm.nih.gov/geo/; TISCH, http://tisch.comp-genomics.org/. Other data used to support the findings of this study are available from the corresponding author upon request.

## RESULTS

### Landscape of genetic variation of methionine regulators in LUAD

**Figure 2:**
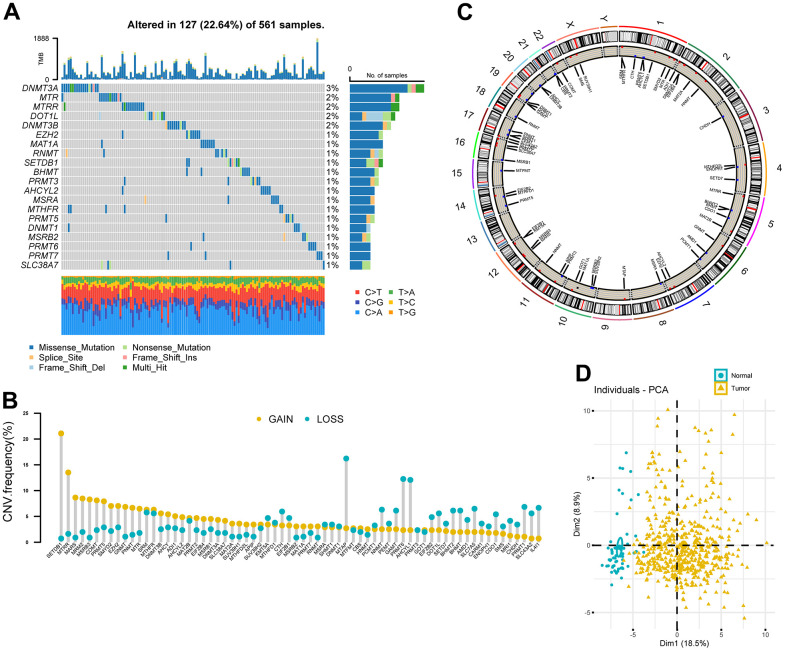
**Landscape of genetic variation of methionine regulators in LUAD.** (**A**) Oncoprint plot showing mutation frequencies of MRGs in 561 patients with LUAD. (**B**) The CNV alteration frequency of 68 MRGs. The column represented the alteration frequency. The deletion frequency, blue dot; The amplification frequency, yellow dot. (**C**) Circos plot showing the location of CNV alteration of MRGs on chromosomes. (**D**) Principal component analysis of 68 MRGs to distinguish tumors from normal samples in the TCGA LUAD cohort.

A total of 68 MRGs were investigated. We first determined the prevalence of somatic mutations of these genes in LUAD. The top 20 frequently mutated genes were shown in Figure 2A. The overall mutation frequency of MRGs was relatively low (127/561, 22.64%) and missense mutation was the most prevalent mutation type. DNA Methyltransferase 3 Alpha (*DNMT3A*) showed the highest mutation frequency, followed by 5-Methyltetrahydrofolate-Homocysteine Methyltransferase (*MTR*), 5-Methyltetrahydrofolate-Homocysteine Methyltransferase Reductase (*MTRR*), DOT1 Like Histone Lysine Methyltransferase (*DOT1L*), and DNA Methyltransferase 3 Beta (*DNMT3B*). The strongest co-occurrence was found between alterations of *SLC38A7* and *MTR*, as well as SET Domain Bifurcated Histone Lysine Methyltransferase 1 (*SETDB1*) and Enhancer of Zeste 2 Polycomb Repressive Complex 2 Subunit (*EZH2*) (Supplementary Figure 1). Analysis of CNV data showed widespread CNV alteration with 68 regulators. *SETDB1* and *MTRR* displayed the most prevalent CNV amplification, whereas the opposite was seen for Methylthioadenosine Phosphorylase (*MTAP*), *PRMT6*, and *AHCYL1* (Figure 2B). The locations of CNV alterations of 68 MRGs on chromosomes were demonstrated in Figure 2C. Interestingly, we observed that *SETDB1*, *PRMT6*, and *AHCYL1* were all located on chromosome 1.

PCA analysis indicated that MRGs’ expression could effectively distinguish tumors from normal samples in the TCGA cohort (Figure 2D). Further analysis in 13 datasets with matched controls (Supplementary Table 2) demonstrated that MRGs were more often up-regulated than down-regulated in LUAD than paired normal. We found *EZH2*, Solute Carrier Family 7 Member 5 (*SLC7A5*), *IL4I1*, *SUV39H2*, and *DNMT3A* were remarkably increased in LUAD, whereas Cysteine Dioxygenase Type 1 (*CDO1*), *MSRB3*, *AHCYL2*, and *MTHFR* were mostly decreased (Figure 1A).

### Prognostic significance of MRGs in LUAD

We next sought to determine the prognostic significance of MRGs in LUAD. A total of 30 datasets including 5024 LUAD patients with survival information were used (Supplementary Table 1). The optimal cutoff for stratifying each gene was determined by the maxstat method. Surprisingly, we found most MRGs were highly prognostic in more than one cohort. Interestingly, we found high expression of the Protein Arginine Methyltransferase (*PRMT*) family genes (*PRMT1*, *3*, *5*, and *6*) were generally associated with a worse survival outcome. Other MRGs that were significant in more than half of the tested datasets were *SLC7A5*, *SRM*, *MTAP*, *RNMT*, *CBS*, *EZH2*, *PRMT1*, *SUV39H1*, *CDO1*, *MSRA*, *GNMT*, *HNMT*, and *AHCYL2*, in which the former eight genes were mostly adverse prognostic indicators and high expression of the latter mostly predicted better prognosis (Figure 1B and Supplementary Table 3). Importantly, a meta-analysis of hazard rations (HRs) and p-values across 30 datasets further confirmed the strong prognostic influence of *SLC7A5* and *CDO1* expression (Supplementary Figure 2).

### Methionine modification patterns mediated by 68 regulators

**Figure 3:**
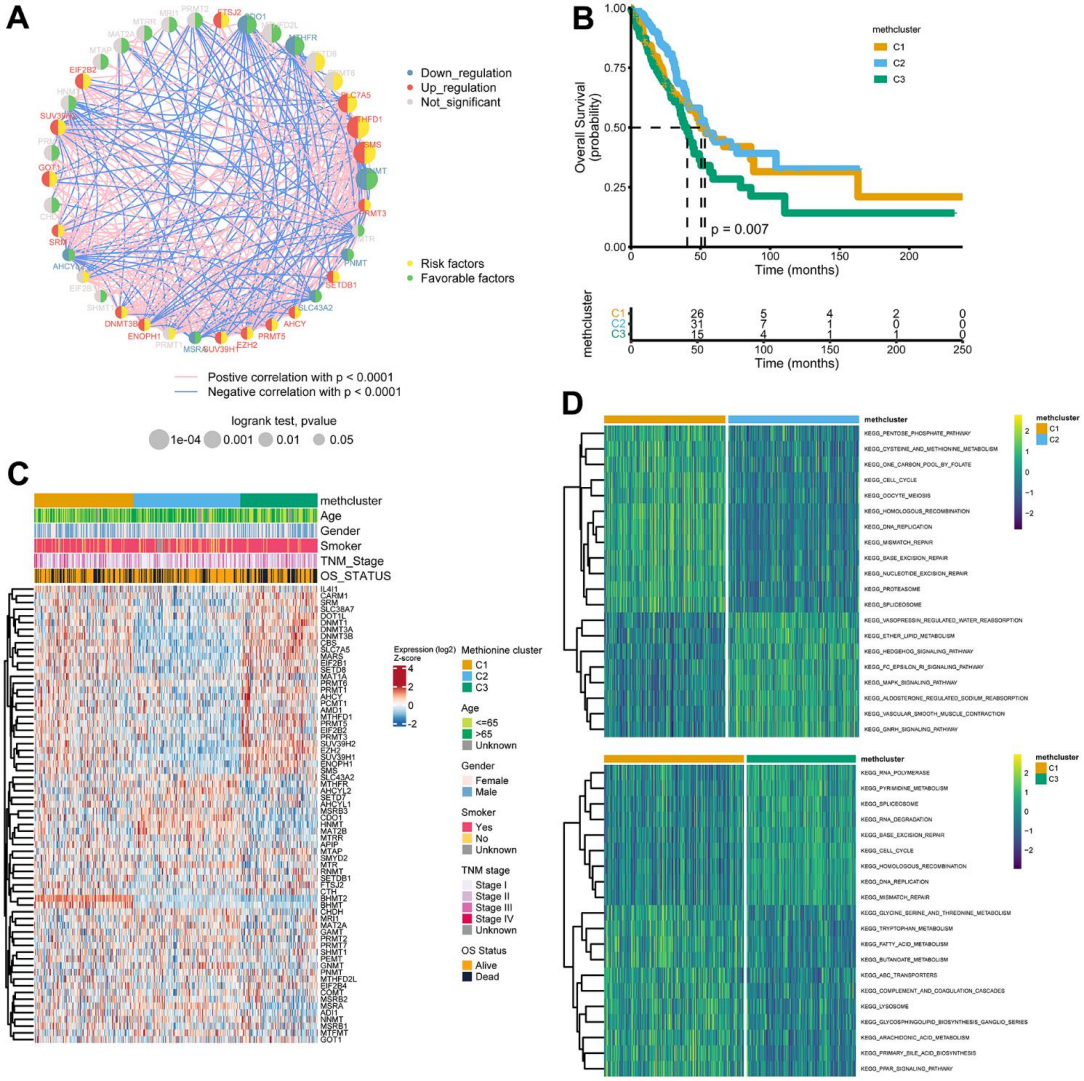
**Patterns of methionine modification and biological characteristics of each pattern.** (**A**) The interaction between key MRGs (Log-rank p-value < 0.05) in LUAD. The circle size represents the impact of each regulator on the prognosis, and the range of values calculated by Log-rank test was p < 0.001, p < 0.01, and p < 0.05, respectively. The color on the left side of the dots depicts differential expression patterns and the color on the right side depicts direction of the prognostic significances. The lines linking regulators showed their interactions, and thickness showed the correlation strength between regulators. Negative correlation was marked with blue and positive correlation with red. (**B**) Survival analysis for the three MRG modification patterns based on 517 patients from the TCGA LUAD cohort. (**C**) Heatmap showing association between the expression of 68 MRGs and clinical characteristics in the TCGA LUAD cohort. The MethClusters, age, gender, smoking status, TNM stage, and survival status were used as patient annotations. (**D**) GSVA enrichment analysis showing the activation states of biological pathways in distinct MRG patterns. The comparison of C1 vs C2 and C1 vs C3 was shown.

In a next step, 39 genes with significant associations with survival in the TCGA cohort (logrank p-value < 0.05, Supplementary Table 5) were illustrated in a network, which showed comprehensive connections, differential expression patterns, and prognostic implications of these genes (Figure 3A). We found that these MRGs showed either positive or inverse correlations, with genes up-regulated in LUAD significantly negatively correlated with those down-regulated in LUAD. Additionally, highly expressed MRGs consistently predicted worse patient outcome, and the opposite was true for the lowly expressed genes.

The above results indicated that cross-talk among the MRGs may play critical roles in the pathogenesis of LUAD. Using the ConsensusClusterPlus package, we grouped LUAD patients with qualitatively different modification patterns based on the expression of 68 MRGs, and three distinct modification patterns were ultimately identified using unsupervised clustering, including 181 cases in cluster 1, 195 cases in cluster 2, and 141 cases in cluster 3. We termed the three clusters as methcluster C1-C3, respectively (Supplementary Figure 3A–3C and Supplementary Table 6). Prognostic analysis for the three methionine subtypes revealed that patients in C3 had the worst prognosis, while patients in C1 and C2 had similar survival rates (Figure 3B). We also observed significant differences in the expression of MRGs among the three clusters. For example, *BHMT2* and *BHMT* were almost exclusively expressed in C1 and genes that adversely impact outcomes, such as *SLC7A5*, *MARS*, *EZH2*, the *DNMT* family (*DNMT1*, *DNMT3A*, and *DNMT3B*), and the *PRMT* family (*PRMT1*, *3*, *5*, and *6*) all showed low expression in C2 but relatively high expression in C3 (Figure 3C). Besides those that exhibited poor outcomes, we found much more smokers were distributed in the C3 cluster (Figure 3C and Supplementary Figure 3D). In addition, we analyzed differences in KEGG pathway scores among 3 methclusters. As expected, we detected differential levels of multiple metabolism pathways, such as KEGG_PENTOSE_PHOSPHATE_PATHWAY, KEGG_CYSTEINE_AND_METHIONINE_METABOLISM, and KEGG_ONE_CARBON_POOL_BY_FOLATE, which were activated in the C1 cluster (Figure 3D). Interestingly, DNA repair pathways including KEGG_DNA_REPLICATION, KEGG_MISMATCH_REPAIR, KEGG_BASE_EXCISION_REPAIR, KEGG_NUCLEOTIDE_EXCISION_REPAIR, KEGG_SPLICEOSOME were also high in C1. In addition, some signaling pathways were differentially enriched (Figure 3D and Supplementary Figure 3E). These results indicated that MRGs might participate in tumor development by impacting various metabolism, DNA repair, and tumor signaling pathways.

### TME and immunological characteristics in distinct MRG modification patterns

**Figure 4:**
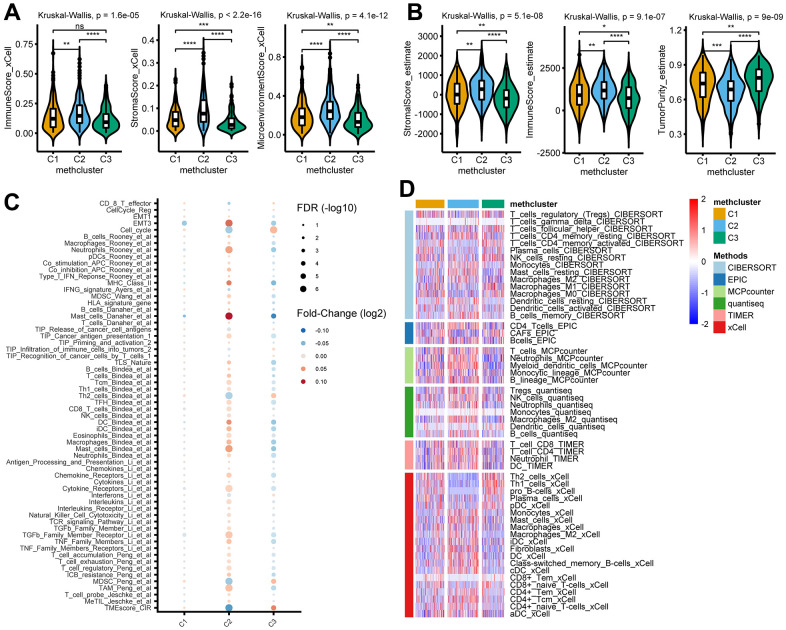
**TME and immunological characteristics in distinct MRG modification patterns.** (**A**, **B**) The differences of indicated TME characteristics (immune score, stromal score, and tumor purity) among three MRG modification patterns. The TME score was evaluated using the xCELL (**A**) and ESTIMATE (**B**) algorithm, respectively. (**C**) The differences of indicated TME-related signatures among three MRG modification patterns. The fold change between each subtype and the remaining samples were compared using the Wilcoxon rank sum test. The color of the dots indicates fold changes (log2) and size indicates the FDR values. (**D**) The differences of indicated immune cells among three MRG modification patterns. The relative cell abundances were calculated using 6 deconvolution algorithms as indicated. *P < 0.05; **P < 0.01; ***P < 0.001; ****P < 0.0001; ns, not significant.

To explore the difference in immune responses among three distinct MRGs modification patterns, we used the xCell (Figure 4A) and ESTIMATE (Figure 4B) algorithms to quantify the overall infiltration of immune cells (Immune Score), stromal components (Stromal Score), and tumor cell purity (Tumor Purity) across three modification patterns. Surprisingly, we found the C2 subtype consistently exhibited the highest TME score, followed by C1 and C3 (Figure 4A, 4B). Conversely, C3 had a higher tumor purity than the C1 and C2 subtypes (Figure 4B), suggesting that C2 subtype tumors are surrounded by more nontumor components (immune cells and stromal cells) while C3 subtype contains more malignant cells. This was consistent with the observed survival differences among the three groups (Figure 3B).

We then analyzed the activity of TME-related signatures among subtypes of methionine metabolism. Our results showed that these pathways were active in C2 but suppressed in C3 (Figure 4C and Supplementary Table 7), further confirming the important role of methionine metabolism in tumor immune regulation. Importantly, further estimating immune infiltration using 6 deconvolution algorithms confirmed the overall enrichment of immune cells in C2 (Figure 4D and Supplementary Table 8).

### Identification of MRG gene subtypes and functional annotation

**Figure 5:**
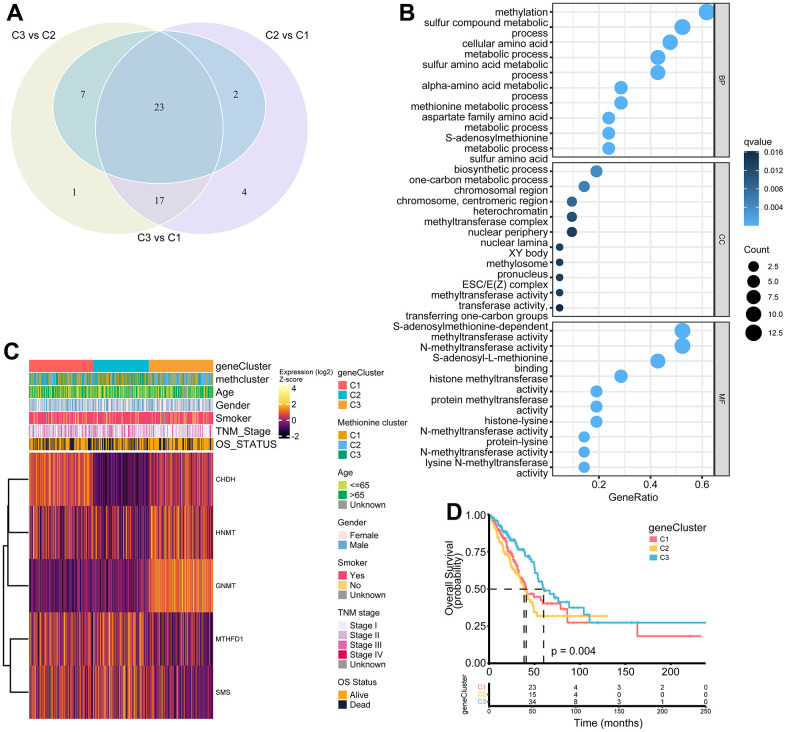
**Construction of MRG gene signatures and functional annotation.** (**A**) Venn diagram showing 23 MRG-related differentially expressed genes (DEGs) among three MRG-clusters. Vs means that the gene expression profiles from one cluster was compared to another cluster. (**B**) Functional annotation for MRG-related genes using GO enrichment analysis. The color depth of the bubbles represents the q value and the size of the bubbles represents the count of genes enriched. (**C**) Heatmap showing association between the expression of core DEGs and clinical characteristics in the TCGA LUAD cohort. The gene clusters, MethClusters, age, gender, smoking status, TNM stage, and survival status were used as patient annotations. (**D**) Survival analysis for the three gene clusters based on 517 patients from the TCGA LUAD cohort.

To further investigate the potential biological features of each methionine regulation pattern, we examined the MRG-related transcriptional expression changes across three methclusters in LUAD. A total of 23 differentially expressed genes (DEGs) that represented the essential distinguishing index of the three subtypes were identified, all of which were in the list of 68 MRGs (Figure 5A and Supplementary Table 9). These MRG subtype-related genes were, as expected, significantly enriched in biological processes that were regulated by methionine metabolism, such as DNA methylation, histone methylation, and N-methyltransferase activity (Figure 5B). KEGG analysis indicated enrichment of amino acids metabolism-related pathways (Supplementary Figure 4A). We then performed univariate Cox regression analysis of the 23 subtype-related genes and determined 5 genes significantly associated with OS (p < 0.05): *HNMT*, *MTHFD1*, *SMS*, *GNMT*, and *CHDH* (Supplementary Table 10). To further validate this regulation mechanism, a consensus clustering algorithm was used to divide patients into three clusters based on the 5 prognostic genes (genecluster C1-C3) (Supplementary Figure 4B). The gene subtype C3 comprises less advanced pathologic stage (stage III/IV) tumors (Figure 5C). The three MRG gene subtypes also showed significant differences in MRG expression (Figure 5C). Further survival analysis indicated significant prognostic differences among the three MRGs gene clusters in LUAD (Figure 5D). Kaplan-Meier curves showed that patients with gene subtype C3 had the best OS, whereas patients in genecluster C1 and C2 showed a relatively poor OS (p = 0.004; Figure 5D).

### Construction of the MethScore and exploration of its clinical relevance

**Figure 6:**
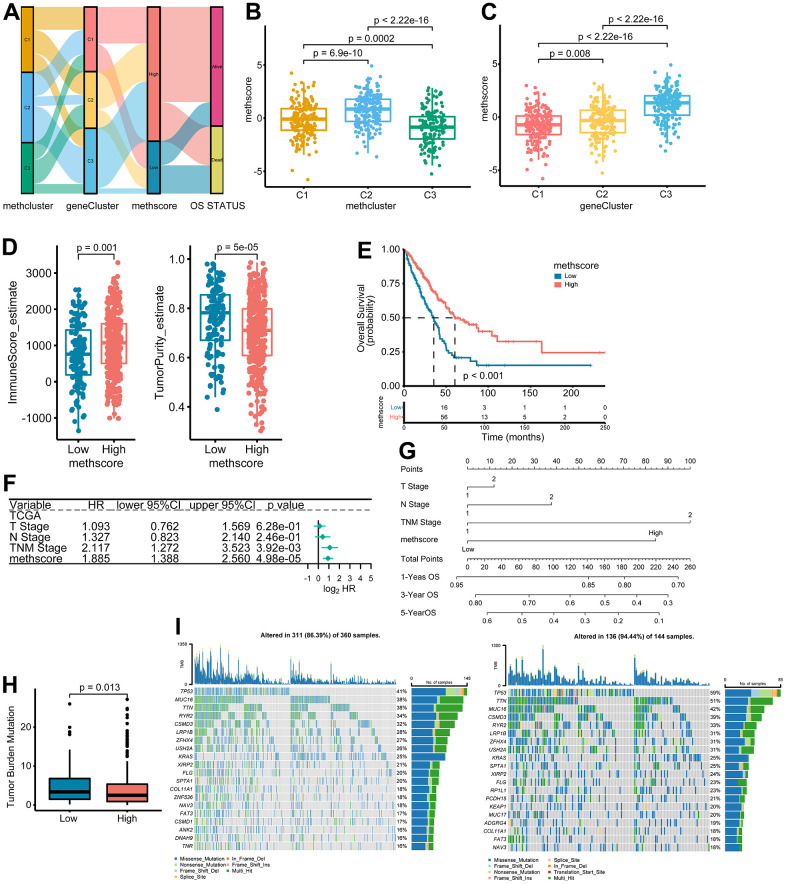
**The association between MethScore with clinical, immunological, and genomic features.** (**A**) Sankey diagram showing the links between MRG cluster, MRG-gene cluster, MethScore, and survival status. (**B**, **C**) Distribution of MethScore in the different MRG clusters (**B**) and gene clusters (**C**). (**D**) Box plots showing the difference of immune score and tumor purity in groups with high or low MethScore. The TME score was evaluated using the ESTIMATE algorithm. (**E**) Kaplan-Meier curves for high and low MethScore patient groups in TCGA LUAD cohort. (**F**) Multivariate analysis of MethScore for OS in the TCGA LUAD cohort. Only variables with p ≤ 0.20 in the univariate analysis were retained. Please see Supplementary Table 5 for the full list of variables. (**G**) Nomogram for predicting 1-, 3-, and 5-year OS for LUAD patients in TCGA cohort. (**H**) Relative distribution of tumor mutation burden (TMB) in patients with high and low MethScore. (**I**) The difference in mutational profiles in TCGA LUAD stratified by high (left panel) versus low MethScore (right panel) subgroups. The top 20 frequently mutated genes were shown.

The above results demonstrated the importance of methionine metabolism in the regulation of TME and prognosis of LUAD. We then constructed a scoring model called MethScore based on the sum of PC1 and PC2 of the five core MRGs. A Sankey diagram was used to visualize the attribute changes of individual patients (Figure 6A). Further analysis revealed significant differences of MethScore among MethClusters and GeneClusters (Figure 6B, 6C). We found MethCluster C2 showed the highest score while C3 had the lowest median score, which indicated that a high MethCluster might be closely linked to immune activation. Accordingly, the high MethScore group showed a higher TME score but lower tumor purity (Figure 6D and Supplementary Figure 5A), once again indicating a strong positive correlation between methionine metabolism and immune infiltration. For GeneCluster, the score is highest in the C3 cluster (Figure 6C). In addition, we showed that the MethScore was positively correlated with the infiltration level of most immune cells, but negatively correlated with the abundance of activated CD4+ T cells (Supplementary Figure 5B).

We then divided the TCGA-LUAD cohort into high or low MethScore groups using the optimal cut-off value obtained by the maxstat method. Patients with low MethScore were significantly associated with a worse prognosis in the TCGA LUAD cohort (p < 0.001, Figure 6E). Furthermore, multivariate Cox regression model analysis considering T stage, N stage, and TNM status confirmed the MethScore as a robust and independent prognostic biomarker for evaluating patient outcomes (p < 0.001, Figure 6F). Then, a nomogram-based survival probability prediction model was constructed based on the MethScore and clinical factors in LUAD. After integrating the total score of clinical factors and locating it on the total point scale, the probability of 1-, 3-, and 5-year survival at each time point could be determined (Figure 6G). As can be seen in the figure, the nomogram predicted well and the concordance index was 0.682 (Figure 6G).

Next, we analyzed the distribution differences of somatic mutations between low and high MethScore groups in TCGA-LUAD cohort. As shown in Supplementary Figure 6, genes were much more frequently mutated in patients with low MethScore than in those with high MethScore. Consistently, the low MethScore group presented a significantly higher TMB and the MethScore was much lower in the high TMB group (Figure 6H and Supplementary Figure 5C). The MethScore and TMB also exhibited a significant negative correlation (Supplementary Figure 5D). The mutational landscape showed that *TP53* (59% vs. 41%) had higher somatic mutation rates in the low MethScore group, whereas *CACNB2* and *KCNT1* were exclusively mutated in the high MethScore subtype (Fisher’s exact test, p < 0.01, Figure 6I and Supplementary Figure 6).

### The role of MethScore in predicting immunotherapeutic benefits

**Figure 7:**
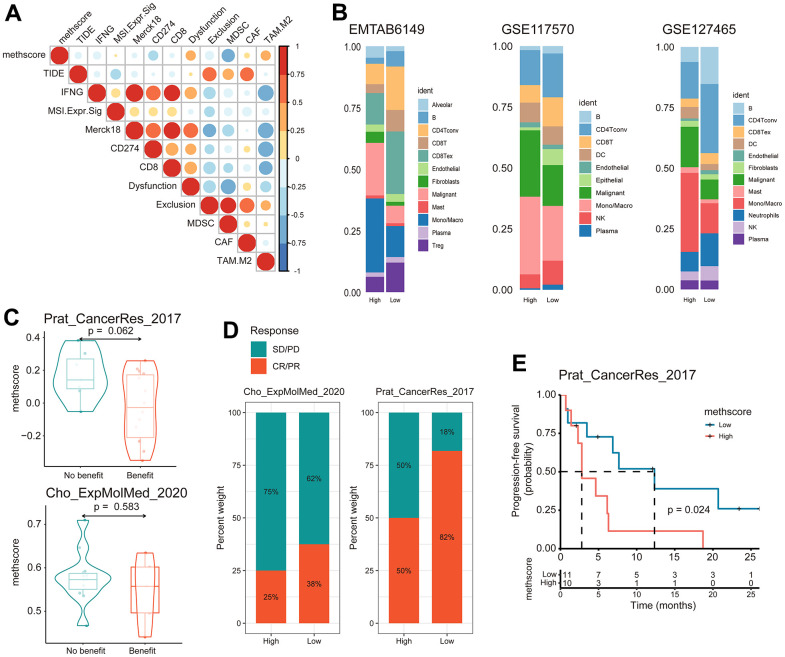
**The MethScore predicts responses to immunotherapy.** (**A**) Correlogram showing the association between MethScore with T cell dysfunction and T cell exclusion signatures in TCGA LUAD cohort, as determined using the tumor immune dysfunction and evasion (TIDE) method. (**B**) Differential composition of immune cell populations in cell samples with high and low methionine activity. The methionine activity was computed in three scRNA-seq data of LUAD (EMTAB6149, GSE117570, and GSE127465). (**C**) Violin plots comparing the MethScore between patients who benefitted and did not benefit from immunotherapy in indicated ICB cohorts. (**D**) The fraction of patients with clinical response to ICB among indicated ICB cohorts between patients with high and low MethScore (as stratified by the median score). CR, complete response; PR, partial response; SD, stable disease; PD, progressive disease. (**E**) Kaplan-Meier curve depicting the PFS of lung cancer patients with high and low MethScore in the indicated ICB cohort.

Our analysis demonstrated that tumor MRG modification patterns play a crucial role in mediating the immune response and were closely linked to patient TMB status. Accumulated evidence revealed TMB status as an emerging biomarker for response to immunotherapy [27, 28]. Therefore, we hypothesized that the difference in tumor MRG modification patterns might be a crucial factor that mediated the clinical response to immunotherapy. Surprisingly, using the TIDE algorithm, we found that MethScore was positively correlated with the T-cell dysfunction score and M2 subtype of tumor-associated macrophages (TAMs) (Figure 7A), despite the higher immune scores observed in the high MethScore group (Figure 6D). We then calculated the methionine metabolism activity at the single-cell resolution using three scRNA-seq datasets derived from lung cancer patients. Interestingly, we found CD8+ T cells were consistently inhibited in samples with higher methionine metabolism activity, whereas the proportion of TAMs and malignant cells was remarkably increased (Figure 7B). These results were consistent with previous findings that methionine metabolism contributes to T cell dysfunction and immune escape in cancer [8].

We next investigated whether the MRG signatures could predict patients’ response to ICB therapy. Using two independent ICB cohorts of lung cancer, we found that patients with a response to ICB had lower MethScore than patients with no response and that the low MethScore group presented a better response to ICB and improved treatment outcomes in terms of progression-free survival (PFS) (Figure 7C–7E). Taken together, our findings strongly suggest that MethScore is associated with the response to immunotherapy.

## DISCUSSION

Increasing evidence demonstrated that methionine metabolism takes on a critical role in protein synthesis, epigenetic regulation, antioxidant defense as well as pro-tumor effects [6]. A recent study has revealed a tumor-initiating capability of methionine cycle activity and two methionine regulators (*MAT2A* and *MTHFR*) in lung cancer [7]. Given the indispensable tumorigenic potential of methionine in lung cancer, we reasoned that an overall characterization of multiple methionine regulators in lung cancer will broaden our understanding of methionine modification and guide the designation of more effective therapies.

In this study, we investigated the genetic and transcriptional heterogeneity of 68 MRGs in LUAD, the most prevalent subtype of lung cancer. We found *DNMT3A* was the most frequently mutated MRGs in LUAD, followed by *MTR*, *MTRR*, *DOT1L*, and *DNMT3B*. Among them, *DNMT3A* and *DNMT3B* are DNA methyltransferases dependent on SAM [29], the major metabolic product of methionine metabolism. While *DNMT3A* and *DNMT3B* are involved in de novo methylation, *DOT1L* is a histone methyltransferase that mediates the methylation of histone H3 lysine 79 (H3K79me) [30]. Therefore, altered methionine metabolism might contribute to LUAD through epigenetic-regulated gene expression patterns. Accordingly, the previous study demonstrated that lung TICs require exogenous methionine for histone methylation, and that the tumorigenic capabilities of TICs were probably endowed through epigenetic alterations [7]. We also observed widespread CNV alterations of certain MRGs in LUAD. For example, *MTAP*, an enzyme involved in the methionine salvage pathway, was found to be deleted in LUAD. Consistently, *MTAP* homozygous deletion occurs frequently in cancers such as glioblastomas [31], melanomas [32], and pancreatic cancer [33]. It should be noted that *MTAP* could also be inactivated by promoter hypermethylation in cancer [34], which indicates epigenetic alterations in MRGs could feed back and modulate methionine metabolism.

Further analysis revealed distinct differential expression patterns and prognostic values of MRGs in LUAD. Overall, genes that were upregulated in LUAD usually predicted worse outcomes; some prominent ones include *EZH2*, *SLC7A5*, *PRMT3*, and *PRMT5*. In line with this, overexpression of *PRMT5* has been implicated in a number of cancer types [35–37]. *MAT2A* and *MTHFR* have been found to be overexpressed in lung tumors [7]; however, they showed no differential expression or even reduced expression in our study. The possible reasons are as follows: First, fewer samples were included in previous studies, whereas we used 13 LUAD datasets to compare MRGs between LUAD and normal samples. Second, gene expressions were assessed at the protein and mRNA level between their study and ours. Therefore, prospective validation is still required.

Unsupervised cluster analysis of the expression values of MRGs identified three distinct modification patterns. Notably, the C2 subtype was characterized by higher immune scores and elevated tumor-infiltrating lymphocytes, corresponding to an immune-inflamed phenotype, while the C3 subtype was characterized by the presence of more malignant cells and a worse survival. Differentially expressed genes were then determined to construct an MRG-related gene cluster, which also had significantly different outcomes. Finally, we established a scoring system to evaluate the MRG modification pattern of individual patients with LUAD—the MethScore. We observed that patients with a higher MethScore were more frequently in the C2 MRG subtype, and accordingly had a higher TME score. Indeed, increasing evidence has shown that metabolism plays a key role in immune regulation [38]. Recently, researchers have uncovered a mechanic link between tumor methionine metabolism and T-cell exhaustion: they showed that SAM and MTA treatment promotes the dysfunction of human CD8+ T cells *in vitro*, and that knockout of *MAT2A* reduces SAM production and suppresses tumorigenesis and T-cell dysfunction in hepatocellular carcinoma (HCC) [8]. Another study by Bian et al. demonstrated that tumor cells could hijack methionine metabolism from CD8+ T cells for their own benefit, thereby impairing T cell survival and function by reducing H3K79me2 levels and inhibiting *STAT5* signaling in CD8+ T cells [9]. Our analysis demonstrated that MethScore was positively correlated with the T-cell dysfunction score and M2 subtype of TAMs. Using scRNA-seq data, we found CD8+ T cells were consistently inhibited in samples with higher methionine metabolism activity, whereas the proportion of TAMs was remarkably increased. In addition, we showed that the MethScore was negatively correlated with the abundance of activated CD4+ T cells. It was previously shown that methionine deprivation limited the immune activation of CD4+ T cells [39]. Therefore, it is possible that methionine was deprived from CD4+ T cells by tumor cells, leading to decreased levels of activated CD4+ T cells. These results further support the view that methionine metabolism contributes to T cell dysfunction and immune escape in cancer [8], suggesting a complicated interaction of MRG modification with tumor immunological characteristics. These findings and ours imply that the MRG modification pattern could be applied in clinical practice to determine immune phenotypes and guide immunotherapy strategies. Importantly, we confirmed the predictive value of the MethScore in two independent ICB cohorts of lung cancer: patients with a low MethScore exhibited robust clinical benefits from ICI therapy.

In summary, we comprehensively evaluated the expression and genomic features of 68 MRGs in LUAD and systematically correlated these modification patterns with clinical and TME characteristics. We identified three MRG modification patterns of LUAD, which were different in terms of clinical outcome, biological processes, and immunological characteristics. Overall, the comprehensive evaluation of MRG modification patterns will enhance our understanding of the relationship between methionine metabolism and immune responses and guide more effective immunotherapy strategies.

## Supplementary Material

Supplementary Figures

Supplementary Table 1

Supplementary Table 2

Supplementary Table 3

Supplementary Tables 4 and 5

Supplementary Table 6

Supplementary Table 7

Supplementary Table 8

Supplementary Tables 9 and 10
