# Supplementary material for: A novel methionine metabolism-related signature predicts prognosis and immunotherapy response in lung adenocarcinoma
Source: Aging (Albany NY). 2023 May 2;15(9):3498–523. doi: 10.18632/aging.204687 (PMC10449287; doi:10.18632/aging.204687)
Supplement: Supplementary Figures [file aging-15-204687-s001.pdf]

SUPPLEMENTARY FIGURES

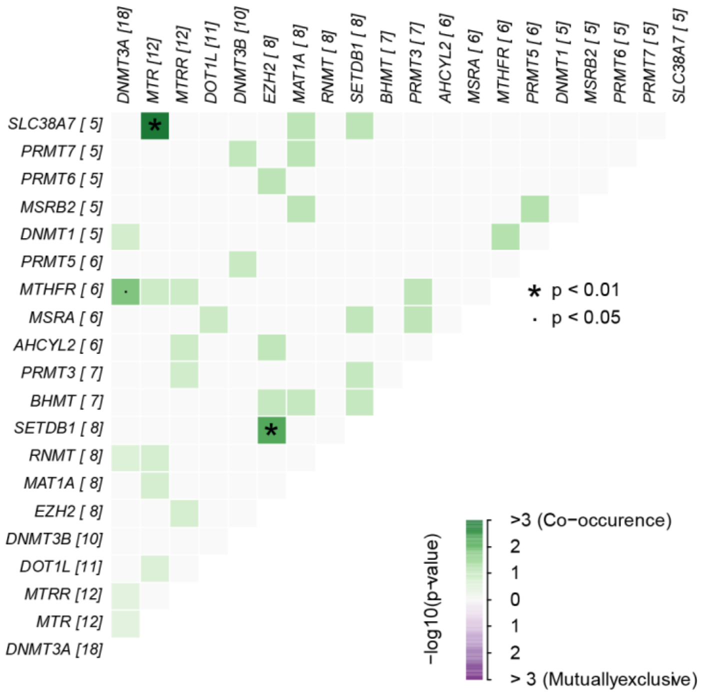

Supplementary Figure 1. The mutation co-occurrence and exclusion analysis for the top 20 frequently mutated MRGs in TCGA LUAD cohort. MRG pairs with co-occurrence or exclusiveness in their mutation pattern were illustrated as a triangular matrix. Green displayed tendency toward co-occurrence, whereas purple showed exclusiveness.

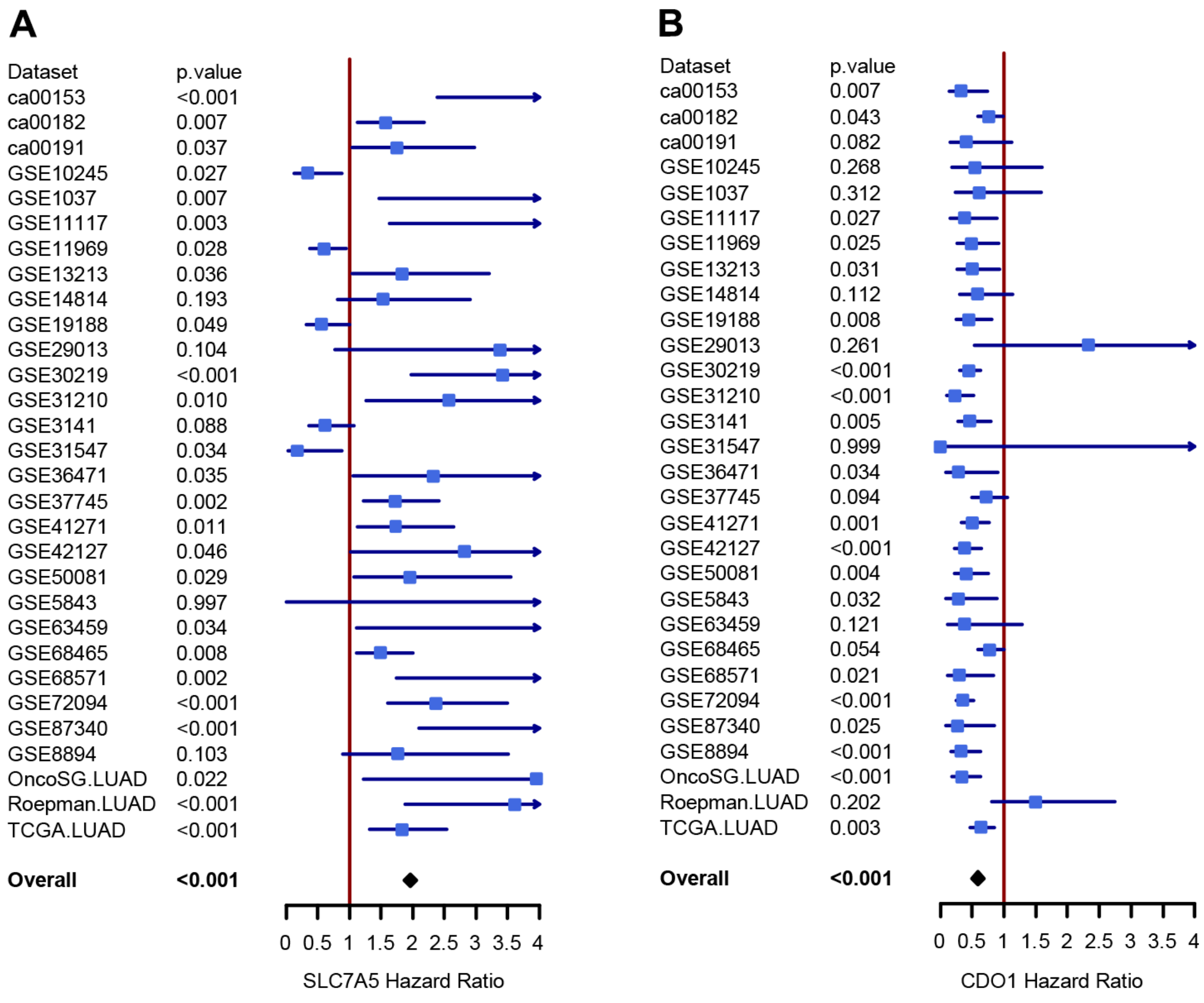

Supplementary Figure 2. Forest plot showing hazard ratios and p-values of SLC7A5 (**A**) and CDO1 (**B**) in each LUAD dataset. The hazard ratios and p-values were combined across datasets with OS information available. The p-values in each dataset were combined using the weighted Z-method.

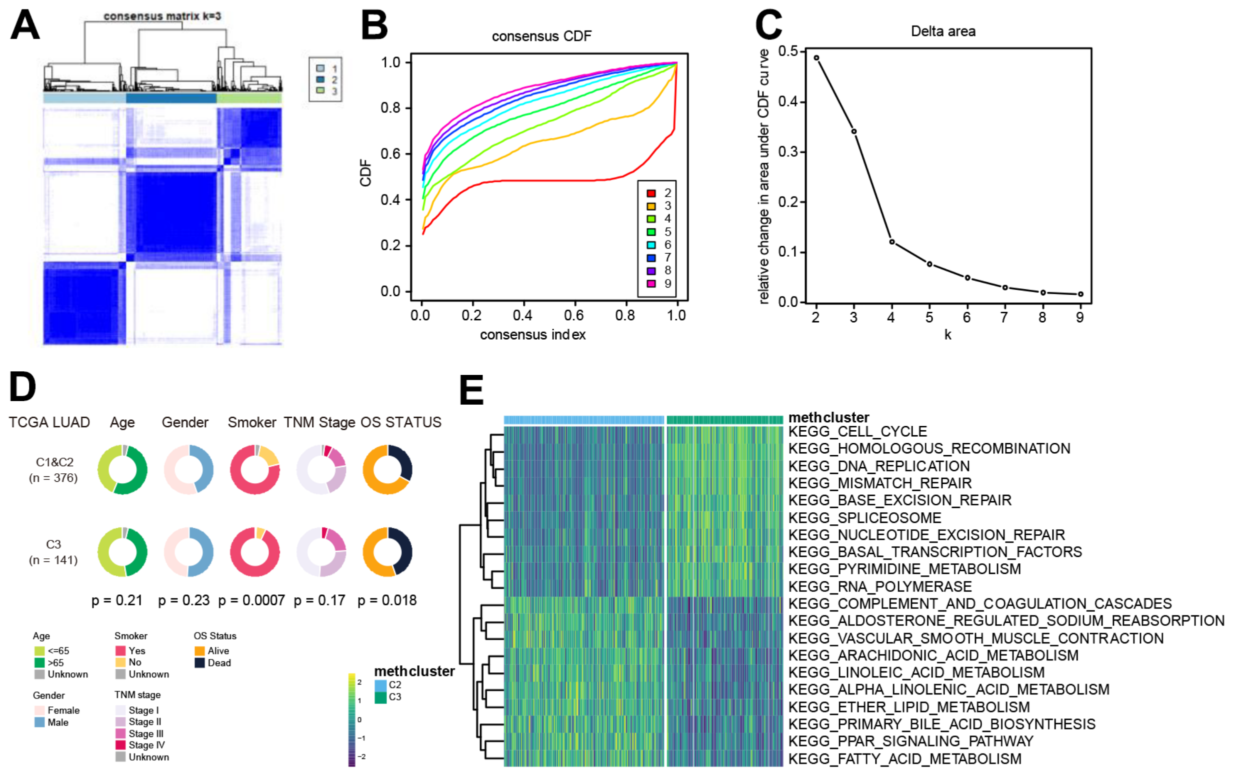

Supplementary Figure 3. Patterns of methionine modification and biological characteristics of each pattern. (**A**) Consensus clustering matrix for k = 3, which was the optimal cluster number in the TCGA LUAD cohort. (**B**) CDF curves of the consensus score (k = 2-9) in the TCGA LUAD cohort. (**C**) Relative change in the area under the CDF curve (k = 2-9) in the TCGA LUAD cohort. (**D**) Pie charts showing the Chi-squared test of clinicopathologic factors between different methclusters (C1&C2 vs C3). (**E**) GSVA enrichment analysis showing the activation states of biological pathways in distinct MRG patterns. The comparison of C2 vs C3 was shown.

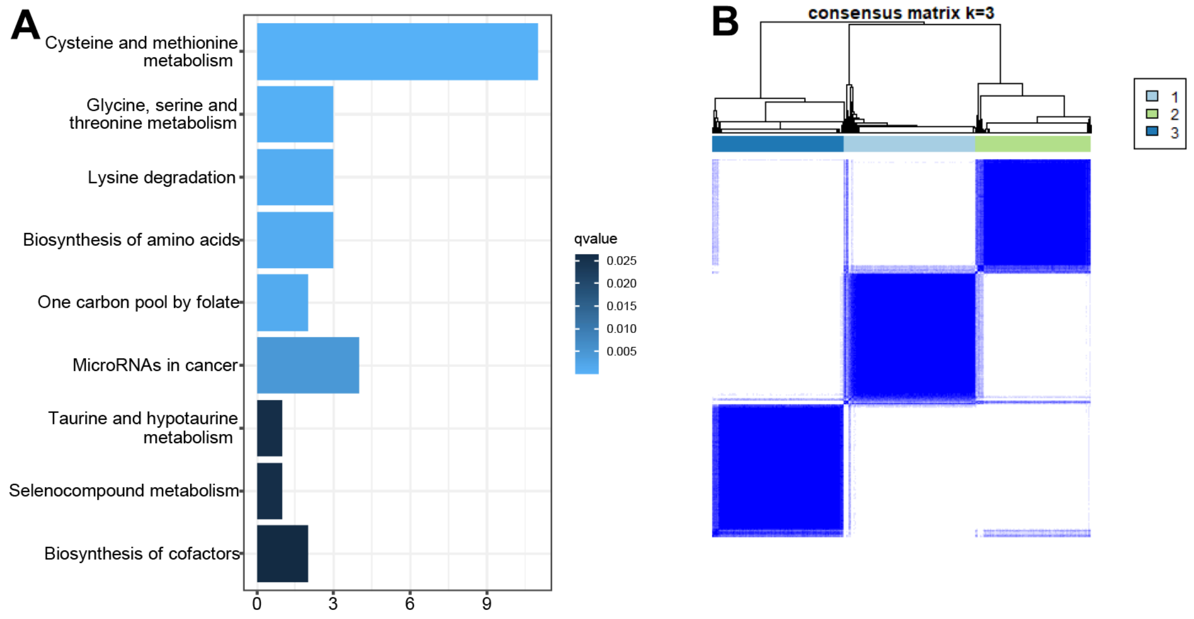

Supplementary Figure 4. Construction of MRG gene signatures and functional annotation. (**A**) Functional annotation for MRG-related genes using KEGG enrichment analysis. The color depth of the bar represents the q value. (**B**) Consensus clustering matrix for k = 3, which was the optimal cluster number in the TCGA LUAD cohort.

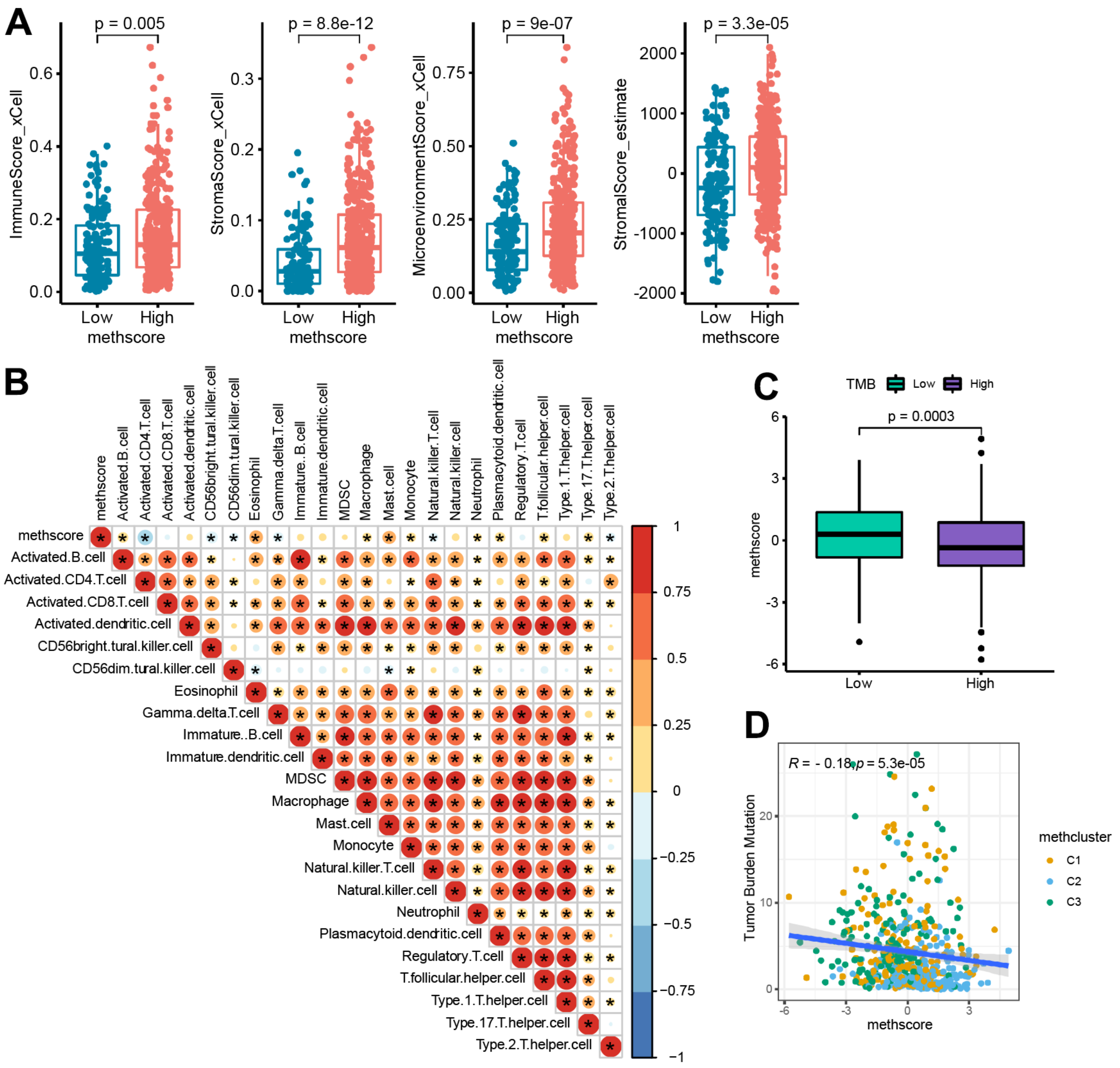

Supplementary Figure 5. The association between MethScore with immunological and genomic features. (**A**) Box plots showing the difference of indicated TME score in groups with high or low MethScore. The TME score was evaluated using the xCell and ESTIMATE algorithm. (**B**) Correlations between MethScore and 22 immune cells using Spearman analysis. The negative correlation was marked with blue and positive correlation with red. The ssGSEA and CIBERSORT algorithms were employed to quantify the relative infiltration levels of the immune cell subsets. (**C**) Relative distribution of MethScore in patients with high and low tumor mutation burden (TMB). (**D**) Scatter plots showing the correlation between MethScore and TMB. P and r values from Spearman correlation analysis were shown.

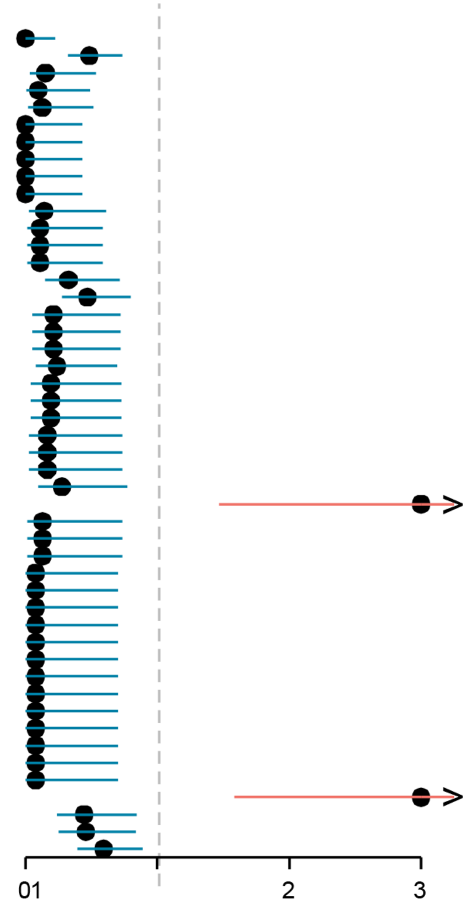

	methscore_low	methscore_high	OR	p-value
POLR2B	8	0	0	***
TP53	85	148	0.485	***
RBBP6	10	4	0.151	***
UBASH3A	8	2	0.095	***
OR6T1	9	3	0.127	***
AMICA1	5	0	0	**
BLM	5	0	0	**
PPP4R4	5	0	0	**
SLC39A3	5	0	0	**
SP4	5	0	0	**
GLG1	8	3	0.144	**
BTBD11	7	2	0.11	**
DNMBP	7	2	0.11	**
TBC1D31	7	2	0.11	**
COL6A6	17	15	0.326	**
RP1L1	33	44	0.469	**
ARHGEF17	9	5	0.212	**
MPO	9	5	0.212	**
PTCH1	9	5	0.212	**
VCAM1	11	7	0.241	**
KCNK18	8	4	0.192	**
STARD13	8	4	0.192	**
TAS2R16	8	4	0.192	**
ACSL1	7	3	0.165	**
KRT1	7	3	0.165	**
USP28	7	3	0.165	**
UGT2B28	11	8	0.276	**
CACNB2	0	15	Inf	**
GPRC5C	6	2	0.129	**
ISOC1	6	2	0.129	**
PCDHGC4	6	2	0.129	**
ALS2CL	5	1	0.078	**
APOBR	5	1	0.078	**
EMILIN1	5	1	0.078	**
GPLD1	5	1	0.078	**
KIAA1841	5	1	0.078	**
KRTAP4-11	5	1	0.078	**
MAGED1	5	1	0.078	**
MICALCL	5	1	0.078	**
OVCH2	5	1	0.078	**
PCM1	5	1	0.078	**
PTEN	5	1	0.078	**
UPP2	5	1	0.078	**
XKR9	5	1	0.078	**
KCNT1	0	16	Inf	**
AHNAK	23	28	0.444	**
SYNE1	26	33	0.459	**
TTN	73	136	0.591	**

Supplementary Figure 6. Forest plot showing the comparison of mutational profiles between patients with high and low MethScore in the TCGA LUAD dataset.
