# Supplementary material for: A novel methionine metabolism-related signature predicts prognosis and immunotherapy response in lung adenocarcinoma
Source: Aging (Albany NY). 2023 May 2;15(9):3498–523. doi: 10.18632/aging.204687 (PMC10449287; doi:10.18632/aging.204687)
Supplement: Supplementary Tables 4 and 5 [file aging-15-204687-s005.pdf]

Supplementary Table 4. A total of 68 methionine-related genes in the study.

Symbol	Source
ADI1	REACTOME_METHIONINE_SALVAGE_PATHWAY
AHCY	Integrative modelling of tumour DNA methylation quantifies the contribution of metabolism
AHCYL1	GOBP_S_ADENOSYLMETHIONINE_CYCLE
AHCYL2	GOBP_S_ADENOSYLMETHIONINE_CYCLE
AMD1	Methionine metabolism in health and cancer: a nexus of diet and precision medicine
APIP	GOBP_S_ADENOSYLMETHIONINE_CYCLE
BHMT	HNF4α regulates sulfur amino acid metabolism
BHMT2	Integrative modelling of tumour DNA methylation quantifies the contribution of metabolism
CARM1	Methionine Cycle
CBS	HNF4α regulates sulfur amino acid metabolism
CDO1	HNF4α regulates sulfur amino acid metabolism
CHDH	Methionine Cycle
COMT	Methionine Cycle
CTH	HNF4α regulates sulfur amino acid metabolism
DNMT1	Methionine Cycle
DNMT3A	Methionine Cycle
DNMT3B	Methionine Cycle
DOT1L	Methionine Cycle
EIF2B1	methionine biosynthetic process
EIF2B2	methionine biosynthetic process
EIF2B4	methionine biosynthetic process
ENOPH1	GOBP_S_ADENOSYLMETHIONINE_CYCLE
EZH2	Methionine Cycle
GAMT	Methionine Cycle
GNMT	Methionine Cycle
GOT1	REACTOME_METHIONINE_SALVAGE_PATHWAY
HNMT	Methionine Cycle
IL4I1	Methionine Cycle
KMT5A	Methionine Cycle
MARS1	Methionine Cycle
MAT1A	Functional genomics reveal that the serine synthesis pathway is essential in breast cancer
MAT2A	Functional genomics reveal that the serine synthesis pathway is essential in breast cancer
MAT2B	Integrative modelling of tumour DNA methylation quantifies the contribution of metabolism
MRI1	GOBP_S_ADENOSYLMETHIONINE_CYCLE
MRM2	Methionine Cycle
MSRA	Functional genomics reveal that the serine synthesis pathway is essential in breast cancer
MSRB2	Functional genomics reveal that the serine synthesis pathway is essential in breast cancer
MSRB3	Functional genomics reveal that the serine synthesis pathway is essential in breast cancer
MTAP	Methionine metabolism in health and cancer: a nexus of diet and precision medicine
MTFMT	Methionine Cycle
MTHFD1	methionine biosynthetic process
MTHFD2L	methionine biosynthetic process
MTHFR	Methionine Cycle
MTR	Integrative modelling of tumour DNA methylation quantifies the contribution of metabolism
MTRR	GOBP_S_ADENOSYLMETHIONINE_CYCLE
NNMT	Methionine Cycle
PCMT1	Methionine Cycle
PEMT	Methionine Cycle

PNMT	Methionine Cycle
PRMT1	Methionine Cycle
PRMT2	Methionine Cycle
PRMT3	Methionine Cycle
PRMT5	Methionine metabolism in health and cancer: a nexus of diet and precision medicine
PRMT6	Methionine Cycle
PRMT7	Methionine Cycle
RNMT	Methionine Cycle
MSRB1	Functional genomics reveal that the serine synthesis pathway is essential in breast cancer
SETD7	Methionine Cycle
SETDB1	Methionine Cycle
SHMT1	Methionine Cycle
SLC38A7	Methionine Transport
SLC43A2	Cancer SLC43A2 alters T cell methionine metabolism and histone methylation
SLC7A5	Cancer SLC43A2 alters T cell methionine metabolism and histone methylation
SMS	Methionine Cycle
SMYD2	Methionine Cycle
SRM	Methionine Cycle
SUV39H1	Methionine Cycle
SUV39H2	Methionine Cycle

Supplementary Table 5. Univariate Cox regression result of 68 MRGs in the TCGA LUAD cohort.

Gene	HR	HR.95L	HR.95H	pvalue	km
GNMT	0.770653673199567	0.691730063992243	0.85858214776486	2.29091935009681E-06	4.34312585895213E-11
SMS	1.59997380500189	1.28972307343853	1.98485723750545	0.00001925742348073	3.19978537044463E-06
MTHFD1	1.55298619967208	1.20824258678777	1.99609429657984	0.000588148762962725	7.10621980848369E-06
SLC7A5	1.16214401544152	1.03781428036945	1.3013684029726	0.00924397641864403	0.00023067836100743
PRMT6	1.04502881925879	0.903252120145111	1.20905914165579	0.553790281018542	0.000360145605322493
SETD8	1.3115365124243	0.977176583125433	1.76030417953773	0.0708874638265721	0.000446833560163173
MTHFR	0.829666989583491	0.68439520258789	1.00577460362331	0.0572581711567003	0.000557942386581689
MTHFD2L	0.801255498150047	0.646913590430834	0.992420599616885	0.0423914546125624	0.000648420026005292
CDO1	0.90667396150655	0.820766291442599	1.00157338458566	0.0537296886823586	0.000754656692648403
FTSJ2	1.25945858865966	0.939333926819406	1.68868161924012	0.123146662703762	0.00156345542258796
PRMT2	0.850473313602593	0.612760237082287	1.18040436271494	0.332875171050306	0.00180835626505016
MRI1	0.744792634505469	0.605053542636515	0.916804926050722	0.00544805032347809	0.00193286688678285
MTRR	0.812049670604717	0.664160997750521	0.992868701659186	0.0423831729418267	0.00269822967008782
MAT2A	0.672109508063635	0.482601786827224	0.936032984459847	0.0187169693158296	0.00281076814837311
MTAP	0.987119703455488	0.849224555243783	1.14740595162175	0.865901860312542	0.00300139848491265
EIF2B2	1.18628989395644	0.905062471493774	1.55490229329751	0.215933349002563	0.00323195959332834
HNMT	0.852339925056659	0.735738238752324	0.987420946174514	0.0332858745036553	0.00400118818421813
SUV39H2	1.17530489111446	0.96782934138114	1.42725739757623	0.103103573289245	0.00406127572596982
PRMT7	0.863596353099823	0.668131524403361	1.11624528082747	0.262691423785109	0.00407651727537472
GOT1	1.12794942989154	0.935342929599871	1.36021760162011	0.207562247424876	0.00907820845871321
CHDH	0.87119042083928	0.770256029426023	0.985351260317546	0.0281743210599072	0.00983152084580197
SRM	1.21729520927679	0.959825987859981	1.54382944957768	0.104851105484751	0.0101433275452283
AHCYL2	0.948795303055893	0.860436082710994	1.04622823843534	0.29194111472637	0.0118618027559739
EIF2B1	1.32374398054339	0.925129641924976	1.89411088631722	0.124968163032936	0.0135173027635095
SHMT1	0.936319279178884	0.778044304162594	1.12679160797359	0.486149075980808	0.0138400109095673
DNMT3B	1.07138304957349	0.96766886464453	1.18621326039569	0.184408590454656	0.0180767859443872
ENOPH1	1.21499866949772	0.896541288960402	1.64657421254184	0.209207693741283	0.0192937079251336
PRMT1	1.21678831771275	0.927739252599752	1.59589432696018	0.156207001668826	0.0198242300625547
BHMT2	0.987005613706406	0.937279397884098	1.0393699932882	0.619961629924726	0.0208361752984604
MSRA	0.90075389573951	0.760694445396504	1.06660116371297	0.225436885053485	0.0209915500735796
SUV39H1	1.20647628240277	0.965510040788739	1.50758144245846	0.0986967195460309	0.0304850281289359
EZH2	1.07018832687955	0.935743234823759	1.22395013115426	0.322002530315364	0.031756259789255
PRMT5	1.25374122623631	0.977286612821823	1.60839925743575	0.0752077275401681	0.0328224229411592
PNMT	0.942052316725147	0.864199959252884	1.02691808527096	0.174970059660026	0.0359420813150034
AHCY	1.15724980990084	0.928180756600161	1.44285163530107	0.194382300100916	0.0363913275545391
SLC43A2	0.775020848530121	0.625486241450518	0.96030460120659	0.01978970803669	0.0378155885981011
SETDB1	1.03991580485008	0.767711365880963	1.40863471512636	0.800441967519744	0.0406897247872571
MTR	0.837662319202561	0.645127869552414	1.08765749261248	0.18372400813831	0.0433203223555916
PRMT3	1.26224788185154	0.960709685876399	1.65842994888228	0.0944908547558041	0.0487200569876071
SLC38A7	1.02712981855446	0.82428979635515	1.27988441544309	0.811512296605085	0.0504606837901911
COMT	1.10609201854468	0.8911652905527	1.37285368545881	0.360347753084245	0.05086365279905
DNMT3A	1.0718282553209	0.872419492655041	1.31681584212205	0.508963765834148	0.0543760864021912
MSRB1	1.08447097578505	0.929761521528458	1.26492360684792	0.301788852847082	0.0588800910034374
MAT2B	0.955210726013453	0.750842252286971	1.21520536212767	0.709094781048809	0.0595681063946989
AHCYL1	0.797335854073813	0.575283452045282	1.10509777733286	0.173859566194053	0.0621675771735962
SETD7	1.12153570105253	0.899381630313009	1.39856350890513	0.308493817249946	0.0699749169663926
RNMT	0.912250344680271	0.688795535317683	1.20819698836382	0.521747118002844	0.0748441879522822
ADI1	0.844259306449333	0.680856777149299	1.04687769946367	0.122941270660216	0.0755773999481508
CTH	1.08136223001509	0.937157454699211	1.24775646465782	0.284094015214203	0.0767162484652339
MSRB3	0.980126981673605	0.864889350367279	1.1107188448981	0.753112165658284	0.0801265738706223
BHMT	0.965067670382737	0.884511725457924	1.0529601605177	0.423972842051891	0.0804562503017044
NNMT	1.02805982644336	0.921039210391936	1.14751575700778	0.621721697970572	0.0838580978235688
CARM1	1.19879783737279	0.874435199280451	1.64347942085616	0.259990712315004	0.08830723086653
SMYD2	1.11243680316344	0.844629832212747	1.46515739065298	0.448278597058724	0.096612136974032

IL4I1	0.987081426232783	0.891538299088591	1.09286358534433	0.802330027511617	0.0989105028293321
PCMT1	1.07842105852689	0.812364502712062	1.43161348826991	0.601452648346337	0.111752369535646
DNMT1	1.03980244140981	0.855718431993289	1.26348700312942	0.694604193705971	0.140169624835835
MTFMT	0.974567568597464	0.705921958051946	1.34544893373622	0.875585364269793	0.140681898896992
EIF2B4	0.894894129881452	0.606530135018971	1.32035567148075	0.57575802407707	0.163034618545632
MARS	1.01135783886	0.801517741246084	1.27613479476249	0.924164549513128	0.166953076127564
APIP	0.939159208885381	0.718805222049156	1.22706401202786	0.645446591024472	0.172723798672296
MSRB2	1.07466166655645	0.855871922753148	1.34938145166726	0.535281279158577	0.196839230136354
PEMT	0.994559763811341	0.809521246989909	1.22189396198121	0.958578224836063	0.196864287312599
DOT1L	1.06995470180367	0.882408066604711	1.29736241908656	0.491661103951679	0.230479863805757
AMD1	0.959073476182539	0.709984972207714	1.29555127041162	0.78535286240328	0.23823840527471
MAT1A	0.995249875728653	0.93216232147243	1.06260711500684	0.886680551859676	0.299018940121025
CBS	0.995552546817452	0.906673332731822	1.09314439687824	0.925569439728456	0.325690373002635
GAMT	0.984400913700314	0.871041652366402	1.11251299666481	0.801141723237438	0.370191752867674
