# Supplementary material for: A novel methionine metabolism-related signature predicts prognosis and immunotherapy response in lung adenocarcinoma
Source: Aging (Albany NY). 2023 May 2;15(9):3498–523. doi: 10.18632/aging.204687 (PMC10449287; doi:10.18632/aging.204687)
Supplement: Supplementary Tables 9 and 10 [file aging-15-204687-s009.pdf]

Supplementary Table 9. A total of 23 differentially expressed genes (DEGs) that represented the essential distinguishing index of the three subtypes.

Symbol
BHMT2
DNMT3B
EZH2
SUV39H2
CBS
MARS
SUV39H1
HNMT
DNMT3A
SETD8
MTHFD1
MAT2B
AHCYL2
MSRA
MSRB3
CDO1
DNMT1
PRMT1
SMS
GNMT
CHDH
AHCY
MTR

Supplementary Table 10. Univariate Cox regression result of 5 subtype-related genes in the TCGA LUAD cohort.

Symbol	HR	HR.95L	HR.95H	pvalue
HNMT	0.852339925056659	0.735738238752324	0.987420946174514	0.0332858745036553
MTHFD1	1.55298619967208	1.20824258678777	1.99609429657984	0.000588148762962725
SMS	1.59997380500189	1.28972307343853	1.98485723750545	0.00001925742348073
GNMT	0.770653673199567	0.691730063992243	0.85858214776486	2.29091935009681E-06
CHDH	0.87119042083928	0.770256029426023	0.985351260317546	0.0281743210599072
